# Abdominal compartment syndrome caused by severe acute gastric distension in a patient with COVID-19: A case report

**DOI:** 10.1097/MD.0000000000034326

**Published:** 2023-07-14

**Authors:** Ki Bum Park, Woo Young Nho

**Affiliations:** a Department of Surgery, School of Medicine, Kyungpook National University, Kyungpook National University Chilgok Hospital, Daegu, Republic of Korea; b Department of Emergency Medicine, School of Medicine, Kyungpook National University, Daegu, Republic of Korea.

**Keywords:** abdominal compartment syndrome, case report, COVID-19, gastric distension, intra-abdominal hypertension, pandemic

## Abstract

The clinical manifestation of coronavirus disease 2019 (COVID-19) ranges from asymptomatic to critical. The gastrointestinal (GI) tract is involved in the early stages of the disease and is recognized as an important entry site for the virus. Consequently, GI manifestations are common in patients with COVID-19; however, the GI presentation of COVID-19 in relation to bowel dilatation has rarely been reported. Here, we report a case of acute severe gastric distension resulting in aortic compression and abdominal compartment syndrome (ACS) in a patient with COVID-19.

**Patient concerns:**

A 72-year-old male presented to the emergency department (ED) with severe abdominal distension. The patient had been confirmed to have COVID-19 5 days prior to the visit.

**Diagnoses:**

Computed tomography revealed critical abdominal distension with severe gastric dilatation, accompanied by compression of the abdominal aorta and distal thrombosis formation.

**Interventions:**

Intravenous fluid resuscitation and support with inotropic agents were initiated immediately, and a large amount of gastric content was evacuated via a nasogastric (NG) tube.

**Outcome:**

Finally, the patient was discharged after 12 days of admission without obvious complications.

**Lessons:**

ACS is critical, which can be caused by a severe degree of acute gastric distension (AGD). Evacuation of the intraluminal contents is the most efficient management strategy. Prognosis is poor, and most previous studies of the transition from AGD to ACS have reported unfavorable outcomes.

## 1. Introduction

The clinical manifestation of coronavirus disease 2019 (COVID-19) ranges from asymptomatic to critical.^[1]^ Gastrointestinal (GI) manifestations are common extrapulmonary presentations of COVID-19, as the GI tract is involved in the early stages of the disease and is an important entry site for the viruses.^[2–4]^ GI manifestations, which include abdominal pain, anorexia, nausea, vomiting, and diarrhea, may appear even earlier than respiratory symptoms.^[4,5]^ Common entities include enterocolitis, ileus, and bowel ischemia due to thromboembolisms associated with coagulopathy.^[2,4]^ In addition, intestinal obstruction without a predisposing cause is rarely reported in patients with COVID-19.^[3,6]^ Previous research has proposed that the angiotensin-converting enzyme 2 receptor mediates viral entry to the GI tract and microthrombosis formation.^[4]^ Several potential theories regarding GI presentation during COVID-19 such as fecal to oral transmission or concept of the microbiota alteration have been reported.^[7]^ Acute gastric distension (AGD) is a condition involving stomach dilatation accompanied by the loss of gastric wall tension, and filling of the GI tract with gas, secretions, and food material, without structural obstruction.^[8]^ AGD may occur in patients with pancreas pathologies such as acute pancreatitis, diabetic neuropathy or ketoacidosis, and eating disorders such as anorexia nervosa and bulimia nervosa, or after trauma or peritoneal surgery.^[9–12]^ AGD is occasionally accompanied by gastric outlet obstruction due to peptic ulcer disease and gastric cancer.^[13]^ However, a preserved muscle tone generally prevents severe dilatation of the stomach. GI presentation in COVID-19 has been found to be related to bowel dilatation such as paralytic ileus, colonic distension, and small bowel obstruction.^[2,5]^ Here, we report a case of acute severe gastric distension resulting in aortic compression and abdominal compartment syndrome (ACS) in a patient with COVID-19.

## 2. Case report

**Figure 1.**
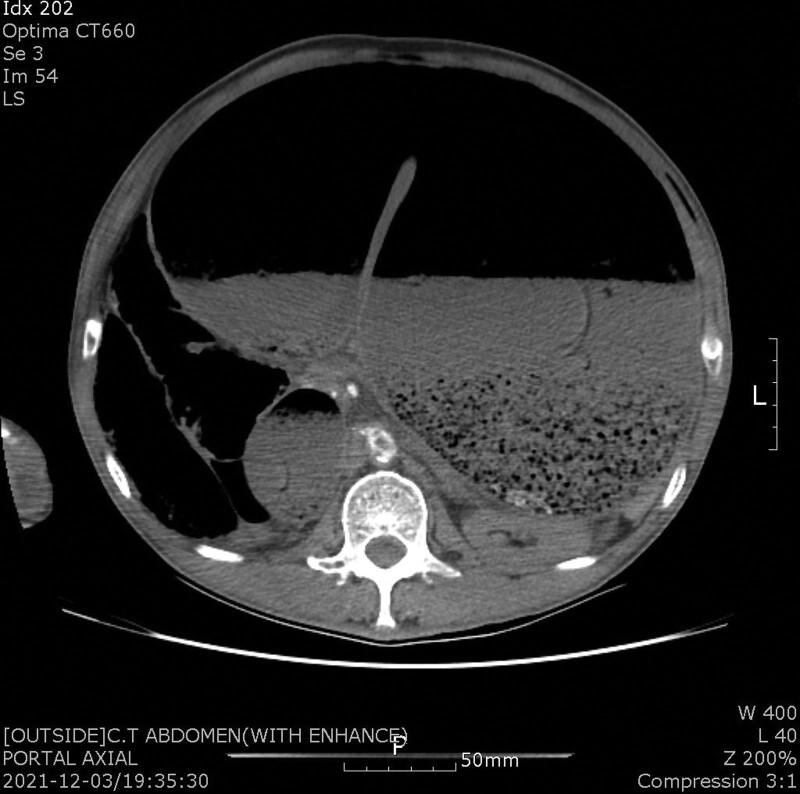
Axial computed tomography image of the patient, showing critical abdominal distension with severe gastric dilatation were noted, in addition to compression of the abdominal aorta and suspicious distal thrombosis.

**Figure 2.**
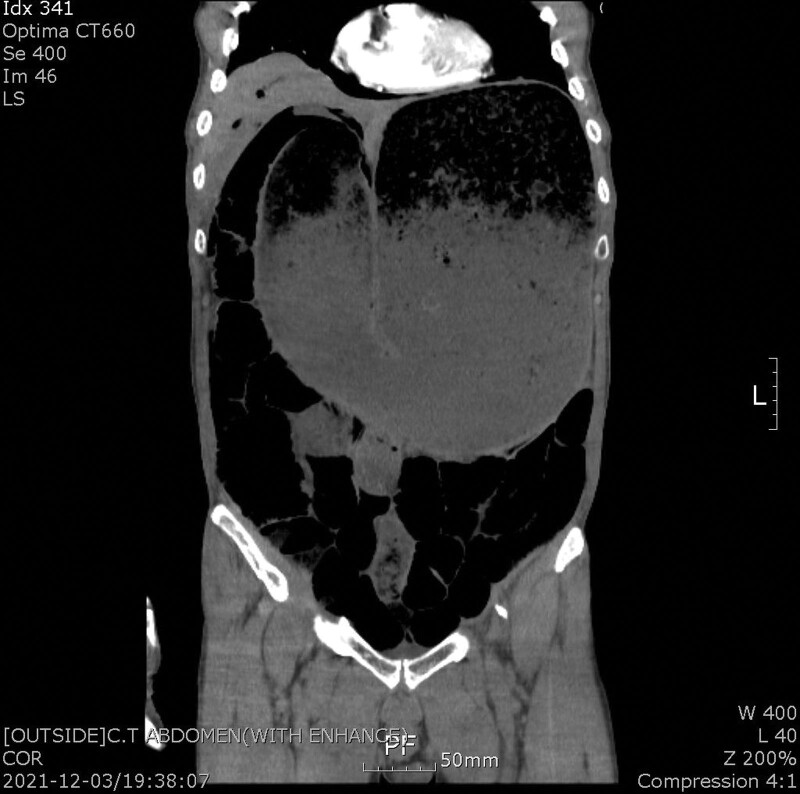
Coronal computed tomography image of the patient showing severe gastric distension resulting in suspicious cardiac deviation and hepatic congestion.

A 72-year-old male presented to our emergency department (ED) with severe abdominal distension. The patient was confirmed to have COVID-19 5 days prior to the ED visit. He was hospitalized in a regional COVID-19 care center, where he began to experience abdominal discomfort the day after admission. He noticed to the staff at the facility; however, his symptom was disregarded, and considered as common GI trouble in COVID-19. Abdominal distension has begun on day 4 and was referred to the ED on the next day. His medical history included diabetes for 15 years, and no relevant surgical history. Further, the patient has no psychiatric history. On arrival at the ED, the patient showed drowsiness with a remarkably distended abdomen. Vital signs included a blood pressure of 75/45 mm Hg and a pulse rate of 119 beats/minutes. Computed tomography revealed critical abdominal distension with severe gastric dilatation (Figs. 1 and 2). In addition, compression of the abdominal aorta and suspected distal thrombosis were noted. Cardiac deviation and hepatic congestion were suspected. Laboratory studies revealed a hemoglobin level of 10.8 g/dL (reference range, 12.0–17.0 g/dL), a white blood cell count of 19,500/mm^3^ (4000–10,500/mm^3^), a platelet count of 59,000/mm^3^ (150,000–450,000/mm^3^), an aspartate aminotransferase level of 125 IU/L (0–40 IU/L), an alanine aminotransferase level of 145 IU/L (0–40 IU/L), a C-reactive protein level of 25.6 mg/L (<5.0 mg/L), and a lactate dehydrogenase level of 951 U/L (300–610 U/L). Blood gas analysis revealed severe respiratory acidosis with a pH of 7.05. Intravenous fluid resuscitation and support with inotropic agents were immediately initiated, and a large amount of the gastric content was evacuated via a nasogastric (NG) tube. Measured intra-abdominal pressure (IAP) reported 22 mm/Hg. The patient was admitted to the intensive care unit for further management. In total, approximately 5 L of gastric contents were evacuated. Moreover, medical therapy included prophylactic antibiotics of third-generation cephalosporin and metronidazole, an antiviral agent for COVID-19 of the intravenous remdesivir for a 3-day protocol, prokinetic agents, and anticoagulation with 1mg/kg of enoxaparin considering the further heparin induced thrombocytopenia development. The anticoagulation was stopped on day 2 because there was no evidence of thrombosis on ultrasonography. And follow-up laboratory study showed no evidence of heparin induced thrombocytopenia development. Esophagogastroduodenoscopy revealed overall nonspecific findings except for chronic atrophic gastritis. The patient initiated oral intake with plain congee on day 7, steamed white rice with vegetables on day 8, and reported no significant gastric distension. The patient was discharged after 12 days of admission. Further, the patient has reported no obvious complication after a follow-up period of 1 month.

## 3. Discussion

ACS is a critical condition with high morbidity and mortality.^[14]^ The concepts of intra-abdominal hypertension (IAH) and ACS have been the focus of research for hundreds of years.^[15]^ In 2004, the World Society of the Abdominal Compartment Syndrome was established to promote research on ACS/IAH with the aim of achieving better outcomes.^[14]^ In 2013, the World Society of the Abdominal Compartment Syndrome published a comprehensive clinical guideline for ACS/IAH that became a practical standard.^[16]^ These guidelines proposed the IAH grade by measuring IAP and suggested relevant medical management strategies based on the 5 principles of therapy. Surgical decompression is strongly recommended when the patient has an IAP > 20 mm Hg, accompanying new-onset organ failure or dysfunction, or is refractory to medical therapies. In addition, patients who presented with severe conditions were regarded as having ACS. Patients with ACS were divided into 2 groups. Primary ACS included peritoneal hemorrhage due to trauma, aortic aneurysm rupture, or mechanical intestinal obstruction. ACS followed by pregnancy, ascites, abdominal sepsis, ileus, and burns was considered secondary.^[17]^ We hypothesize that transient superior mesenteric artery syndrome due to prolonged poor oral intake with particular viral infection as the potential cause of the current manifestation. Also, gastric hypo-motility due to diabetic conditions may contribute to the current situation. Other possible causes, described above, were excluded given the personal history or initial evaluation. A severe degree of AGD may trigger the development of IAH and ACS as in the present case. Among the therapeutic principles of AGD treatment, evacuating the intraluminal content is the most efficient. Prior research has shown that NG tube insertion and initiation of a gastroprokinetic agent may be effective, while rectal tube insertion or colonoscopic decompression are probably less effective for AGD.^[18]^ The prognosis of ACS combined with AGD is poor. Most prior studies of the progression of AGD to ACS have reported unfavorable outcomes.^[9,19–21]^ Decompression is insufficient in cases where the food materials or other particles inside the stomach are larger than the hole at the tip of the NG tube. Autopsy studies have revealed that gastric volvulus or direct compression of the upper GI tract by the distended stomach is a crucial cause of death in ACS with AGD.^[19,20]^

Another limitation included the delayed surgical consultation. Due to higher demand and shortage of resources, longer ED stays in the institution for surgical patients were reported during that period.^[22]^ Notably, the patient was included for candidate of surgical decompression with the high IAP. Accordingly, the result may be concluded unfavorable, in case of non-surgical method of decompression were inadequate.

## 4. Conclusion

Herein, we report a case of acute severe gastric distension resulting in aortic compression and ACS in a patient with COVID-19. Although the patient presented with critical manifestation, timely management and proper treatment resulted in a favorable outcome. Still, the clear causal relation between ACS and COVID-19 are lacked with limited information. However, the physicians should be aware of fatal unusual clinical manifestations even in pandemic.

## Author contributions

**Conceptualization:** Ki Bum Park.

**Methodology:** Woo Young Nho.

**Writing – original draft:** Ki Bum Park.

**Writing – review & editing:** Woo Young Nho.
